# A Crafty Approach for Learning the Topographical Anatomy of the Cranial Nerves

**DOI:** 10.1007/s40670-022-01529-6

**Published:** 2022-03-04

**Authors:** Lisa Quinn

**Affiliations:** Leicester Medical School, The University of Leicester, Lancaster Road LE 1 7HA, George Davies Centre, UK

**Keywords:** Cost-effective, Anatomical education, Cranial nerves, Brainstem, Teaching methods, Neuroanatomy

## Abstract

Certain neuroanatomical relationships can be complex for students to visualise and understand. This article describes creation of a simple and cheap model to help students more easily learn the relationships of the cranial nerves to each other and to the different regions of the central nervous system (forebrain and brainstem).

One of the challenges for learning anatomy is understanding the spatial relationships and orientations between structures. This is particularly true in smaller, more compact areas within the body, where structures are small and overlapping, for example, towards the base of the skull [1]. The use of simple “models” made from easily sourced cheap materials can be used for helping students better understand this aspect of anatomy providing an alternative or adjunct tool to the use of cadaveric/digital resources which may not be widely available for all students. In engaging students in the *construction* of these simple models, explicit attention can be given to the spatial orientations and relationships of the structures to be learnt.

In this article, an activity involving creation of a “model” demonstrating cranial nerve (CN) and brainstem anatomy is described. Required resources include a toilet roll tube and a collection of pipe cleaners (the thinnest you can source) of 3 different colours. The activity is aimed at students (in any discipline studying neuroanatomy) who have some prior knowledge of the CNs and brainstem. However, students’ knowledge may still be in the process of being developed. The activity works well with groups of students collaborating but can easily be completed independently. The activity begins with a retrieval practice exercise, with students bringing prior learnt information *to mind*, one of the most effective learning approaches for durable learning [2]. This includes recalling the names, numbers and functions of the 12 CNs, whether each CN is mixed, motor or special sensory, and their basic topographical relationship to the brainstem. This recalled knowledge will then *applied* to *create* the model, pushing students to higher levels of cognitive challenge [3].

The next part of the activity, students select 12 coloured pipe cleaners, representing each CN; the colour indicates whether the CN is mixed, purely motor or special sensory. The toilet roll tube is used to create the brainstem, as shown in Fig. 1. A 1–2 cm strip is cut from the back of the tube, before laying it flat. Students segment the tube with drawn horizontal lines, representing the parts of the brainstem: midbrain, pons and medulla. A small section at the very top represents part of the forebrain (this can be shaded to indicate clearly that this is not part of the brainstem).

**Fig. 1:**
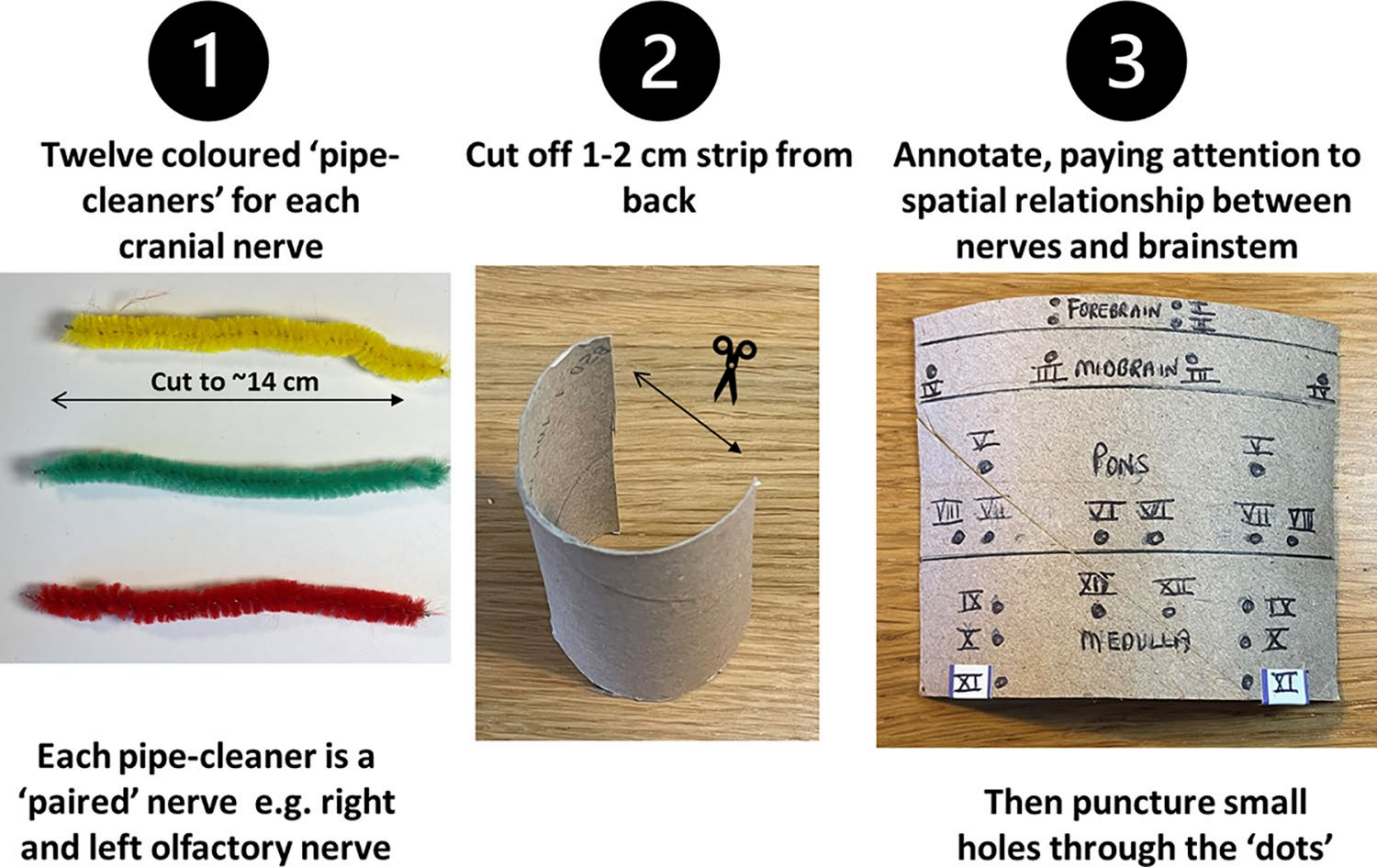
Each cranial nerve is assigned a coloured pipe cleaner; the toilet roll tube is then annotated to demonstrate the forebrain and brainstem regions and the relationship of the cranial nerves to these areas

Using a pencil, students mark with dots the topographical relationship of the 12 paired cranial nerves to each “segment” of the tube. Attention is given to key relational features of the CNs to the various parts of the brainstem, e.g., CN IV arising from the dorsum of the midbrain, several CNs arising at the pontomedullary junction. Using a sharp instrument (e.g., a pin), holes are punctured through each pencilled dot (the tip of a pencil can help widen the hole further). Each CN “pipe cleaner” is then threaded through the relevant holes in the forebrain and brainstem levels. Both ends of the pipe cleaner are passed through the holes to represent the paired CNs. The model is complete once all CNs have been threaded through the tube (Fig. 2). The CNs can be cut and shortened further, if necessary, labels added and the model placed upright, bending the tube slightly to return its natural curvature. The model, if made to a slightly smaller scale, can also be placed into the foramen magnum and the relationship between the brainstem, cranial nerves and skull base can be appreciated.

**Fig. 2:**
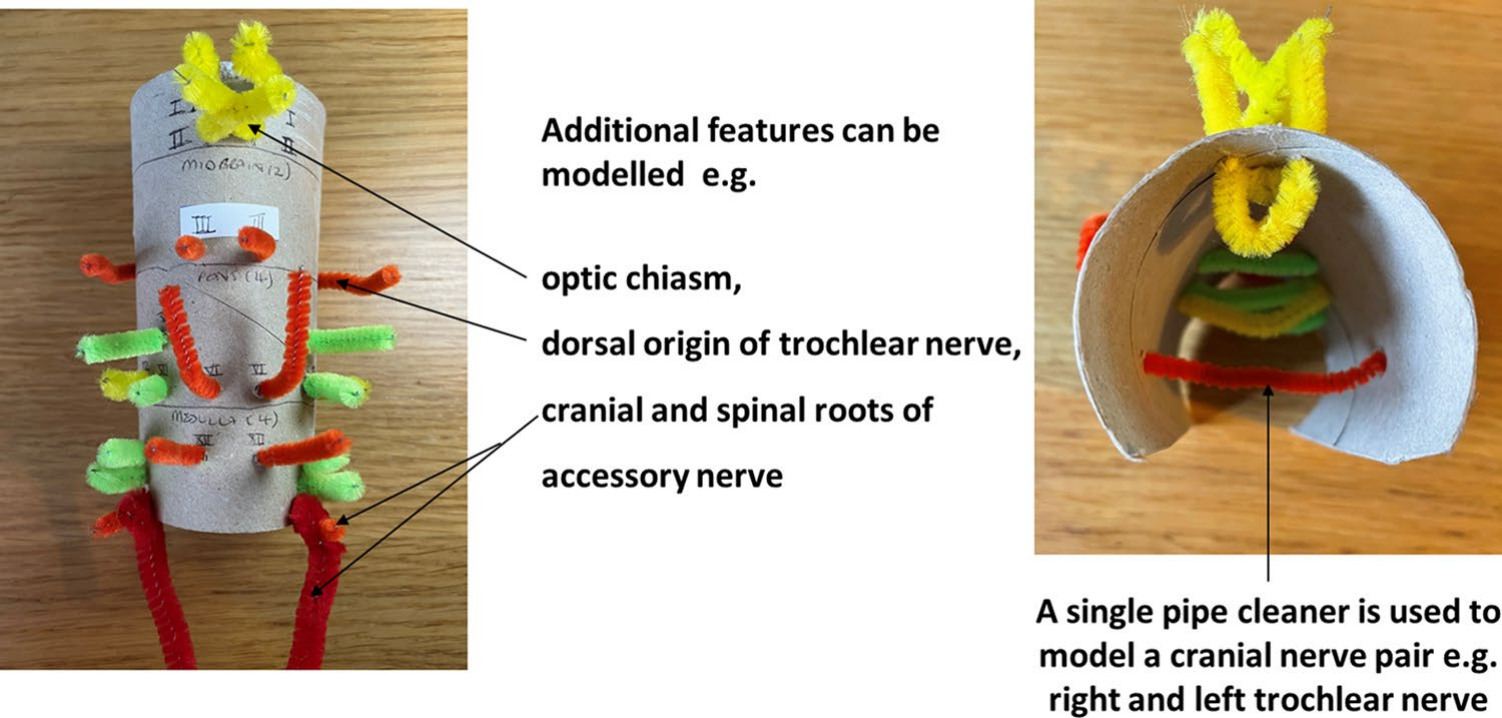
Completed model with all 12 cranial nerves and their topographical relationship to the forebrain and brainstem; a superior view of the model demonstrates the use of each pipe cleaner to model each cranial nerve pair

Anonymised qualitative student feedback received during routine teaching evaluations following introduction of this activity to 32 Year 2 medical students indicated that creation of the model was beneficial for learning and supported a deep level of understanding.

*It gave me an appreciation of the origins (midbrain, pons *etc.*) of the cranial nerves which I hadn’t appreciated before. This has been really helpful in neuro especially with regards to lesions (whether or not they effect sensation of the face). Having something physical to take away is a good reminder!*

However, it was highlighted that most benefit was gained when they had prior knowledge of this anatomy to draw on rather than using this activity to learn the anatomy for the first time. If students have good prior knowledge, minimal instruction is sufficient. More explicit guidance and regular feedback through each step is necessary if prior knowledge is limited or poorly recalled.

In conclusion, this activity can be used or adapted for use by other educators involved in teaching this area of anatomy to support and reinforce students’ understanding of the cranial nerves and their relationship to the central nervous system.
